# Differential expression profiling of components associated with exoskeletal hardening in crustaceans

**DOI:** 10.1186/1471-2164-9-575

**Published:** 2008-12-01

**Authors:** Anna V Kuballa, Abigail Elizur

**Affiliations:** 1 Department of Primary Industries and Fisheries (DPI&F), Animal Science, Bribie Island, Queensland 4507, Australia; 2 School of Integrative Biology, The University of Queensland, St Lucia, Queensland 4072, Australia; 3 Faculty of Science, Health and Education, University of the Sunshine Coast, Maroochydore, Queensland 4558, Australia

## Abstract

**Background:**

Exoskeletal hardening in crustaceans can be attributed to mineralization and sclerotization of the organic matrix. Glycoproteins have been implicated in the calcification process of many matrices. Sclerotization, on the other hand, is catalysed by phenoloxidases, which also play a role in melanization and the immunological response in arthropods. Custom cDNA microarrays from *Portunus pelagicus *were used to identify genes possibly associated with the activation pathways involved in these processes.

**Results:**

Two genes potentially involved in the recognition of glycosylation, the C-type lectin receptor and the mannose-binding protein, were found to display molt cycle-related differential expression profiles. C-type lectin receptor up-regulation was found to coincide with periods associated with new uncalcified cuticle formation, while the up-regulation of mannose-binding protein occurred only in the post-molt stage, during which calcification takes place, implicating both in the regulation of calcification. Genes presumed to be involved in the phenoloxidase activation pathway that facilitates sclerotization also displayed molt cycle-related differential expression profiles. Members of the serine protease superfamily, trypsin-like and chymotrypsin-like, were up-regulated in the intermolt stage when compared to post-molt, while trypsin-like was also up-regulated in pre-molt compared to ecdysis. Additionally, up-regulation in pre- and intermolt stages was observed by transcripts encoding other phenoloxidase activators including the putative antibacterial protein carcinin-like, and clotting protein precursor-like. Furthermore, hemocyanin, itself with phenoloxidase activity, displayed an identical expression pattern to that of the phenoloxidase activators, i.e. up-regulation in pre- and intermolt.

**Conclusion:**

Cuticle hardening in crustaceans is a complex process that is precisely timed to occur in the post-molt stage of the molt cycle. We have identified differential expression patterns of several genes that are believed to be involved in biomineralization and sclerotization and propose possible regulatory mechanisms for these processes based on their expression profiles, such as the potential involvement of C-type lectin receptors and mannose binding protein in the regulation of calcification.

## Background

Arthropods periodically undergo cyclic molting (shedding of the exoskeleton), a process that is essential for growth, metamorphosis and reproduction. Molting in crustaceans is divided into a number of stages: pre-molt, ecdysis, post-molt, and intermolt [1]. Partial digestion and reabsorption of the old exoskeleton, and the initial deposition of a new cuticle occur during pre-molt. At ecdysis the old exoskeleton is shed, followed by the expansion, continued deposition, and hardening of the new cuticle in post-molt, until the crab enters intermolt [2]. The crustacean intermolt cuticle is divided into four layers; the outermost is the epicuticle, beneath which lies the exocuticle (both are deposited pre-ecdysially), and below these are the principle and membranous layers of the endocuticle (deposited post-ecdysially) [3-5]. The crustacean cuticle is biphasic and is composed of an intertwining organic phase, consisting of chitin rods embedded in a protein matrix, and a mineral phase mainly comprised of calcium salts (in the calcified regions of the cuticle) [5]. The epicuticle layer is calcified but is characterised by an absence of chitin, instead its organic matrix is composed of lipids and proteins. The exocuticle, and principle layer of the endocuticle, contain both calcium salts and a matrix of chitin and protein, whereas the membranous layer contains the chitin-protein matrix but remains un-calcified [4,5].

Hardening of the new crustacean exoskeleton is a multifaceted process of which there are two main components: mineralization and sclerotization. Mineralization of a pre-formed organic matrix primarily involves the deposition of calcium salts, principally CaCO_3 _and Ca_3_(PO_4_)_2 _[6], which are deposited as calcite, vaterite, and hydroxyapatite [7] in all layers of the crustacean cuticle except the outer epicuticle and the inner membranous layer. The pre-ecdysial layers calcify after ecdysis and the post-ecdysial principle layer calcifies as it is laid down [3]. Sclerotization refers to the hardening of the organic matrix itself, and requires the enzymatic oxidation of phenols, or catechols, which then interact with the cuticular proteins and chitin, to crosslink and harden them [8,9].

Calcification of the crustacean exoskeleton is most clearly evident in the hard carapace of crabs and lobsters, and is strictly regulated to occur only after ecdysis. A proposed model for the nucleation of calcium in the cuticular matrix of *Callinectes sapidus *suggests that nucleation sites are attached to the chitin-protein matrix. Larger macro-molecules shield these sites from calcium ions and prevent crystal growth [10]. Following ecdysis, an alteration to the macro-molecules occurs to free the nucleation sites and allow the initiation of calcification; possible alterations include deglycosylation, enzymatic cleavage or a change in solubility [10]. Glycosylation of proteins is important in a variety of cellular processes including the regulation of protein function, enzyme trafficking, and immunity [11]. Glycoproteins also appear to play an important role in the promotion and inhibition of crystal growth during the calcification of many matrices (teeth [12]; mollusc shells [13,14]; sea urchin [15]). Glycoproteins associated with calcification have been extracted from the calcified exoskeletons of crustaceans [16-21]. Furthermore, marked post-ecdysial changes in the lectin-binding characteristics of mannose rich *C. sapidus *cuticular glycoproteins coincided with the onset of mineralization [21], pointing to an association of glycosylation levels and the regulation of mineralization.

Sclerotization is catalysed by phenoloxidases (POs). In addition, POs play a role in melanization and the immunological response in arthropods [22-24]. POs are produced by proteolytic cleavage from an inactive precursor, prophenoloxidase (proPO), by a cascade of serine proteases which are themselves under tight control [25]. The arthropod proPO system is activated by a series of pattern-recognition proteins that are capable of binding to polysaccharides (and other compounds typically associated with microorganisms), proteinases, and other factors such as proteinase inhibitors [23]. POs are copper-containing proteins that catalyse the oxidation of phenolic substances. They include tyrosinases, which catalyse the hydroxylation of monophenols to o-diphenols and the oxidation of o-diphenols to o-quinones (o-hydroxylation of phenolic compounds followed by an oxidation of the diphenolic products), and catecholoxidases, which catalyse only the oxidation, but not the hydroxylation step [22,24,26]. Quinones can then be further polymerised non-enzymatically to the black pigment, melanin [27] which is involved in cuticle pigmentation. Studies into the crab *Cancer magister *have demonstrated that the primary source of PO activity comes from the hemocytes, which accumulate in the connective tissue below the epidermis during pre-molt, leading to the suggestion that these hemocytes are stimulated to release proPO immediately before or after ecdysis, and that the proenzyme moves across the epidermis into the layers of the newly secreted exoskeleton, facilitating sclerotization [22]. Hemocyanin, an oxygen transport protein found in the hemolymph of crustaceans and molluscs, also exhibits PO activity [28-32]. Strong sequence similarities exist between arthropod POs and hemocyanins, especially at the amino acid residues forming the copper binding sites [33]. The dual role of hemocyanin, as an oxygen carrier and metaboliser of phenolic compounds, raises the question of how these processes are regulated. One model suggests that amino acids prevent the phenolic substances, but not molecular oxygen, from reaching the copper atoms of the active site [29]. Removal of residues either through conformational change or proteolysis may allow the phenols to enter and become metabolised [29].

Crustaceans represent an excellent model for studying the mineralization and sclerotization of an extracellular matrix, as hardening of the newly formed exoskeleton is under temporal regulation restricted to the post-molt stage. This enables the study of tightly defined stages, and the isolation of specific biochemical and molecular processes involved in exoskeletal hardening. Many types of cuticle proteins have been shown to be integrated into the exoskeleton of crustaceans [34,35], however the molecular mechanisms involved in cuticle hardening remain poorly understood. In this study we have used a microarray approach to identify a suite of genes potentially associated with the activation pathways and enzyme cascades involved in the mineralization and sclerotization of the crustacean cuticle.

## Results

### Microarray expression analysis

**Figure 1:**
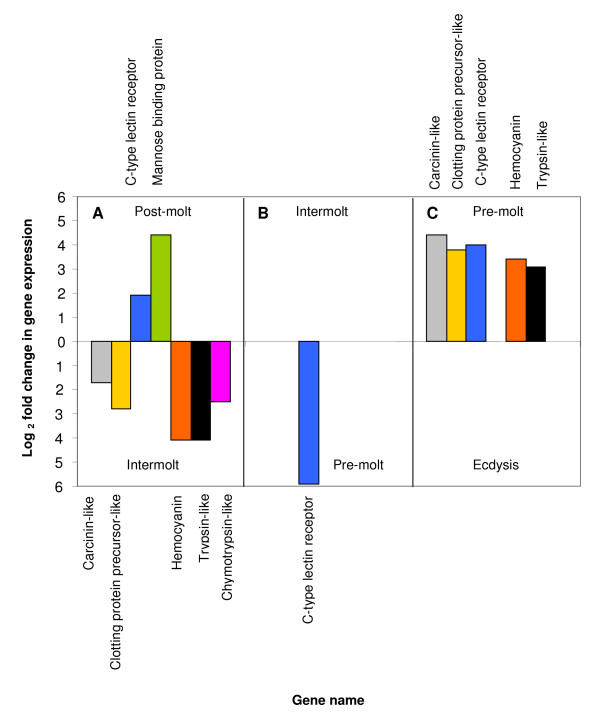
**Differential expression profiles of genes potentially associated with cuticle hardening across molt stages**. Where no bar is evident, no differential gene expression was observed.

Dual channel microarray hybridisation experiments were used to identify differentially expressed genes across consecutive molt stages during the molt cycle of *P. pelagicus*. The temporal differential expression patterns for genes relevant to cuticle hardening are summarised in Tables 1, 2, 3, 4 (multiple values for different transcripts indicate individual cDNA expression levels within a contig), comparing post-molt and intermolt, intermolt and early pre-molt, and late pre-molt and ecdysis. A graphic representation of the log_2 _fold change in gene expression across molt stage comparisons is depicted in Figure 1. 280 unique transcripts were identified within the scope of the microarray experiments described, however only those potentially associated with cuticle hardening will be discussed in this paper.

**Table 1:** The list of transcripts associated with cuticle hardening that are up-regulated in post-molt (Cy3) crabs when compared against crabs in the intermolt stage (Cy5).

**Clone IDs (Accession #)**	**BLAST results**	**Score (Bits)**	**E value**	M *	t *	P-value*	Adjusted P-value*
**PpMBP1 (EF120990)**	AAX55747 mannose-binding protein *Pacifastacus leniusculus*	110	6e-23	5 4.666 4.627 4.571 4.2 4.126 3.888	9.536 8.122 9.365 7.937 8.445 8.573 8.984	0 0 0 0 0 0 0	0.006 0.01 0.007 0.011 0.009 0.008 0.007
**PpMBP2** **(EF120991)**	AAX55747 mannose-binding protein *P. leniusculus*	104	8e-21	3.865	5.7	0.002	0.032
**PpCTLR** **(EF120992)**	AAP58737 C-type lectin receptor *Paralabidochromis chilotes*	46.6	8e-04	2.015 1.845	5.564 5.722	0.002 0.002	0.035 0.032

M = *log*_2 _fold change in expression, t = *t*-statistic, and the *P *value = *t*-distribution. The adjusted *P *values were calculated using the False Discovery Rate (FDR) procedure. Positive values of M and t indicate up-regulation in the Cy3 sample (* multiple values represent several cDNAs all forming one contig).

**Table 2:** The list of transcripts associated with cuticle hardening that are up-regulated in intermolt (Cy5) crabs when compared against crabs in the post-molt stage (Cy3).

**Clone IDs** **(Accession #)**	**BLAST results**	**Score (Bits)**	**E value**	M *	t *	P-value*	**Adjusted** P-value*
PpHEM7 **(EF110537)**	AY861677 *Cancer magister *hemocyanin AAW57890 hemocyanin *C. magister*	290 455	6e-75 1e-126	-5.147 -4.716 -4.432	-10.466 -17.112 -14.02	0 0 0	0.004 0.002 0.002
PpHEM3 **(EF110533)**	AF249297 *Callinectes sapidus *hemocyanin subunit mRNA AAW57891 hemocyanin subunit 3 *C. magister*	807 432	0.0 1e-119	-4.849 -3.951	-16.719 -14.337	0 0	0.002 0.002
PpTRY1 **(EF120993)**	AB205408 *Seriola quinqueradiata *mRNA for trypsinogen CAA75310 trypsin *Litopenaeus vannamei*	75.8 318	2e-10 2e-85	-4.655 -4.434 -4.193 -4.154	-9.19 -13.924 -11.15 -12.959	0 0 0 0	0.007 0.002 0.004 0.003
PpHEM1 **(EF110531)**	AY861676 *C. magister *hemocyanin subunit 1 AAW57889 hemocyanin subunit 1 *C. magister*	103 335	9e-19 1e-90	-4.473 -4.393 -4.112	-9.907 -15.061 -15.684	0 0 0	0.005 0.002 0.002
PpTRY2 **(EF120994)**	AAL67442 trypsin *Paralithodes camtschaticus*	62.0	1e-08	-4.473	-10.021	0	0.005
PpHEM4 **(EF110534)**	AF249297 *C. sapidus *hemocyanin subunit mRNA AAF64305 hemocyanin subunit *C. sapidus*	870 472	0.0 6e-132	-4.402 -4.295 -4.027 -3.954 -3.784 -3.75 -3.707 -2.331	-14.753 -14.847 -15.206 -14.946 -14.715 -14.898 -12.568 -6.86	0 0 0 0 0 0 0 0.001	0.002 0.002 0.002 0.002 0.002 0.002 0.003 0.018
PpHEM2 **(EF110532)**	AY861677 *C. magister *hemocyanin subunit 2 AAW57890 hemocyanin subunit 2 *C. magister*	176 160	8e-41 1e-47	-4.273	-9.162	0	0.007
PpHEM5 **(EF110535)**	AF249297 *C. sapidus *hemocyanin subunit AAW57893 hemocyanin subunit 5 *C. magister*	327 195	3e-86 2e-89	-4.18	-12.698	0	0.003
PpHEM6 **(EF110536)**	AF249297 *C. sapidus *hemocyanin subunit mRNA AAW57891 hemocyanin subunit 3 *C. magister*	807 432	0.0 1e-119	-4.155 -4.083 -3.999 -3.977 -3.946 -3.869 -3.518	-16.298 -11.88 -15.838 -14.718 -15.306 -14.029 -13.331	0 0 0 0 0 0 0	0.002 0.003 0.002 0.002 0.002 0.002 0.003
PpTRY3 **(EF120995)**	Y15041 PVTRYPSN3 *Penaeus vannamei *trypsin gene CAA75311 trypsin *Litopenaeus vannamei*	63.9 253	6e-07 3e-66	-4.119 -4.017 -3.972 -3.246 -3.225	-10.005 -11.911 -12.269 -8.569 -10.838	0 0 0 0 0	0.005 0.003 0.003 0.008 0.004
PpHEM8 **(EF110538)**	AF249297 *C. sapidus *hemocyanin subunit AAF64305 hemocyanin subunit *C. sapidus*	95.6 109	3e-16 3e-22	-3.41	-11.878	0	0.003
PpCTRY1 **(EF120996)**	CAA71673 Chymotrypsin BII *L. vannamei*	32.7	4.9	-2.935	-9.127	0	0.007
EF120998	AAD16454 clotting protein precursor *Pacifastacus leniusculus *(crayfish)	38.9	0.10	-2.83	-8.196	0	0.01
PpCTRY2 **(EF120997)**	HAY12273 *H. armigera *(cotton bollworm – arthropod) mRNA for putative serine protease (chymotrypsin-like protease) CAA71672 Chymotrypsin BI *Litopenaeus vannamei*	50.1 159	0.008 6e-38	-2.433 -2.021	-8.984 -6.564	0 0.001	0.007 0.021
**EF120999**	CAD20734 carcinin *Carcinus maenas *(antibacterial protein)	71.2	2e-11	-1.696	-5.514	0.002	0.036

M = *log*_2 _fold change in expression, t = *t*-statistic, and the *P *value = *t*-distribution. The adjusted *P *values were calculated using the False Discovery Rate (FDR) procedure. Negative values of M and t indicate up-regulation in the Cy5 sample (* multiple values represent several cDNAs all forming one contig).

**Table 3:** A list of transcripts associated with cuticle hardening that are up-regulated in early pre-molt (Cy5) crabs when compared against crabs in the intermolt stage (Cy3).

**Clone IDs** **(Accession #)**	**BLAST results**	**Score (Bits)**	**E value**	M *	t *	P-value*	**Adjusted** P-value*
PpCTLR **(EF120992)**	AAP58737 C-type lectin receptor *Paralabidochromis chilotes*	46.6	8e-04	-6.000 -5.963 -5.667	-32.791 -11.628 -31.811	0 0 0	0.012 0.029 0.012

M = *log*_2 _fold change in expression, t = *t*-statistic, and the *P *value = *t*-distribution. The adjusted *P *values were calculated using the False Discovery Rate (FDR) procedure. Negative values of M and t indicate up-regulation in the Cy5 sample (* multiple values represent several cDNAs all forming one contig).

**Table 4:** A list of transcripts associated with cuticle hardening that are up-regulated in late pre-molt (Cy3) crabs when compared against crabs in ecdysis (Cy5).

**Clone IDs**	**BLAST results**	**Score (Bits)**	**E value**	M *	t *	P-value*	Adjusted P-value*
Carcinin **(EF120999)**	CAD20734 carcinin *Carcinus maenas *(antibacterial protein)	71.2	2e-11	4.769 4.52 4.366 4.358 4.32 4.209 4.176	13.448 14.309 17.315 10.443 16.541 12.818 15.864	0 0 0 0 0 0 0	0.01 0.01 0.01 0.015 0.01 0.01 0.01
PpCTLR **(EF120992)**	AAP58737 C-type lectin receptor *Paralabidochromis chilotes*	46.6	8e-04	4.178 3.938 3.929 3.831	16.607 14.064 14.175 11.36	0 0 0 0	0.01 0.01 0.01 0.013
PpHEM1 **(EF110531)**	AY861676 *Cancer magister *hemocyanin subunit 1 AAW57889 hemocyanin subunit 1 *C. magister*	103 335	9e-19 1e-90	3.953 3.942 3.842	12.659 10.385 10.802	0 0 0	0.01 0.015 0.015
**EF120998**	AAD16454 clotting protein precursor *Pacifastacus leniusculus *(crayfish)	38.9	0.10	3.764	14.388	0	0.01
PpHEM3 **(EF110533)**	AF249297 *Callinectes sapidus *hemocyanin subunit mRNA AAW57891 hemocyanin subunit 3 *C. magister*	807 432	0.0 1e-119	3.714 3.26	11.321 10.152	0 0	0.013 0.015
PpHEM4 **(EF110534)**	AF249297 *C. sapidus *hemocyanin subunit mRNA AAF64305 hemocyanin subunit *C. sapidus*	870 472	0.0 6e-132	3.688 3.654 3.247 3.214 3.207 3.163 3.16 2.075	10.413 12.337 9.711 10.436 9.52 11.682 8.346 7.813	0 0 0 0 0 0 0.001 0.001	0.015 0.01 0.016 0.015 0.017 0.012 0.025 0.029
PpHEM7 **(EF110537)**	AY861677 *C. magister *hemocyanin AAW57890 hemocyanin *C. magister*	290 455	6e-75 1e-126	3.628 3.548 3.187	8.628 10.607 7.652	0.001 0 0.001	0.022 0.015 0.03
PpHEM6 **(EF110536)**	AF249297 *C. sapidus *hemocyanin subunit mRNA AAW57891 hemocyanin subunit 3 *C. magister*	807 432	0.0 1e-119	3.572 3.565 3.429 3.37 3.297 3.211 2.979	9.35 13.009 10.33 9.005 7.107 9.707 9.247	0 0 0 0 0.001 0 0	0.018 0.01 0.015 0.02 0.037 0.016 0.018
PpTRY3 **(EF120995**)	Y15041 PVTRYPSN3 *Penaeus vannamei *trypsin gene CAA75311 trypsin *Litopenaeus vannamei*	63.9 253	6e-07 3e-66	3.474 3.278 3.024 2.892	8.198 12.823 6.46 6.616	0.001 0 0.002 0.002	0.025 0.01 0.048 0.047
PpHEM5 **(EF110535)**	AF249297 *C. sapidus *hemocyanin subunit AAW57893 hemocyanin subunit 5 *C. magister*	327 195	3e-86 2e-89	3.339	12.09	0	0.01
PpHEM2 **(EF110532)**	AY861677 *C. magister *hemocyanin subunit2 AAW57890 hemocyanin subunit 2 *C. magister*	176 160	8e-41 1e-47	3.33	10.225	0	0.015
PpTRY1 **(EF120993)**	AB205408 *Seriola quinqueradiata *mRNA for trypsinogen CAA75310 trypsin *L. vannamei*	75.8 318	2e-10 2e-85	3.275 3.122	12.784 7.468	0 0.001	0.01 0.033
PpTRY2 **(EF120994)**	AAL67442 trypsin *Paralithodes camtschaticus*	62.0	1e-08	2.769	6.565	0.002	0.047

M = *log*_2 _fold change in expression, t = *t*-statistic, and the *P *value = *t*-distribution. The adjusted *P *values were calculated using the False Discovery Rate (FDR) procedure. Positive values of M and t indicate up-regulation in the Cy3 sample (* multiple values represent several cDNAs all forming one contig).

### Comparison of differential expression between post-molt (Cy3) and intermolt (Cy5)

Two unique mannose-binding protein transcripts were up-regulated by a combined average of 8.8-fold in the post-molt stage when compared to intermolt. The mannose-binding protein transcripts contain a C-type lectin domain and a signal peptide. Mannose-binding protein transcripts were not found to be differentially expressed in any other molt stage comparison. Additionally the mRNA coding for the C-type lectin receptor was up-regulated 3.8-fold in post-molt, this transcript was also up-regulated in early pre-molt when compared to intermolt and in late pre-molt when compared to ecdysis. The C-type lectin receptor contains a C-type lectin domain, a signal peptide and transmembrane region, indicating that it may be secreted across the membrane. The level of up-regulation of these transcripts is detailed in Table 1.

Eight unique transcripts of hemocyanin were isolated during this study, corresponding to subunits 1–3 and 5 of *C. magister*. The combined average level of up-regulation observed for hemocyanin during the intermolt stage when compared against post-molt was 8.2-fold. Trypsin-like and chymotrypsin-like transcripts were also found to be up-regulated in intermolt compared to post-molt, at 8.2 and 5-fold respectively. In addition trypsin-like transcripts were up-regulated in late pre-molt when compared with ecdysis. Interestingly two transcripts coding for proteins similar to those typically associated with immune function in other species, the carcinin-like (antibacterial protein) and one similar to clotting protein precursor, displayed molt cycle-related differential expression profiles. The carcinin-like transcript was up-regulated by 3.4-fold in intermolt when compared to post-molt while the one similar to clotting protein precursor displayed a 5.6-fold increase in expression. Carcinin-like and clotting protein precursor-like were also up-regulated in late pre-molt when compared against ecdysis. Details of these results are shown in Table 2.

### Comparison of differential expression between intermolt (Cy3) and early pre-molt (Cy5)

The C-type lectin receptor was the only gene associated with cuticle hardening that was differentially expressed between intermolt and early pre-molt. Up-regulation, by 11.8-fold, in early pre-molt was observed for the C-type lectin receptor transcript. Details of expression levels are depicted in Table 3.

### Comparison of differential expression between early pre-moult (Cy3) and late pre-moult (Cy5)

Microarray analysis indicates that no statistically significant differential gene expression can be observed between the early pre-moult and late pre-moult stages.

### Comparison of differential expression between late pre-molt (Cy3) and ecdysis (Cy5)

Transcripts for carcinin-like and clotting protein precursor-like displayed an up-regulation of 8.8 and 7.6-fold respectively, in late pre-molt when compared against crabs in ecdysis. The C-type lectin receptor was 8-fold up-regulated in late pre-molt. Seven of the eight hemocyanin transcripts isolated in this study, also displayed up-regulation, by a combined average of 6.8-fold, in late pre-molt when compared to ecdysis. Furthermore transcripts for a trypsin-like gene were up-regulated by 6.2-fold in late pre-molt. Details of these expression levels are presented in Table 4.

### Comparison of differential expression between ecdysis (Cy3) and post-moult (Cy5)

Microarray analysis found no statistically significant differential gene expression between ecdysis and the post-moult stage of the moult cycle.

## Discussion

Cuticle hardening is integral to the molting process in arthropods. The principle components of hardening in the newly formed crustacean exoskeleton are calcification and sclerotization. Calcification involves the incorporation of calcium salts into both the pre- and post-ecdysial layers of the crustacean exoskeleton after molting. Sclerotization involves the 'tanning' of cuticular proteins by sclerotizing compounds, such as phenols, which become oxidized and interact with proteins and chitin in the cuticular matrix, to cross-link and harden them. Many proteins involved in exoskeletal calcification and sclerotization have been isolated, yet these processes still remain poorly understood in crustaceans. The objective of this study was to examine the process of cuticle hardening using a holistic, gene expression profiling approach. A *P. pelagicus *cDNA microarray developed in our laboratory was used to identify genes involved in the hardening of the crustacean exoskeleton during the molting process, and to trace their expression profiles across the entire molt cycle. The microarray chips contain 5000 cDNAs derived from both the entire animal and individual organs such as the brain, eyestalk, MO and Y-organ from all molt cycle stages. Thus the arrays were designed to study global gene expression profiles of transcripts relevant to the molting process, across the entire molt cycle. Microarray technology offers the potential to examine the expression patterns of many genes simultaneously, thus gaining a more comprehensive understanding of gene function, interaction, and regulation.

Glycoproteins have been implicated in the control of calcification in diverse structural matrices of both vertebrates [36,37] and invertebrates [16]. Glycosylation of cuticular proteins is thought to be a regulator of biomineralization of the crustacean exoskeleton [16,21]. It has been proposed that glycoproteins act as pre-molt inhibitors of calcification in the exocuticle, and that deglycosylation post ecdysis may remove this barrier to mineralization [10,16]. While the extent of cuticular protein glycosylation can not be determined by gene expression data, as glycosylation is an extensive modification to protein structure that occurs post-translationally, we have, through microarray expression analysis, identified two genes, C-type lectin receptor and mannose-binding protein, that may be relevant to the molt cycle-related alteration of cuticular glycoproteins described elsewhere. In our study, transcripts of the C-type lectin receptor display an up-regulation during early pre-molt (11.8-fold) when compared to intermolt, an up-regulation in late pre-molt of 8-fold when compared to ecdysis, and an up-regulation of 3.8-fold in post-molt when compared to intermolt (Figure 1). The temporal expression patterns of the C-type lectin receptor of *P. pelagicus *point to its involvement in the inhibition of calcification of the crustacean exoskeleton. The high level of up-regulation observed in the pre-molt stages, and up-regulation also in post-molt, coincide with formation of new cuticle. The newly deposited cuticle must remain uncalcified in the pre-molt stage conferring the pliability required for ecdysis as well as growth. The cuticle then hardens following ecdysis and expansion. On the other hand the membranous layer, arthrodial membranes, gills and the gut remain uncalcified at all times. Therefore a mechanism facilitating the regulation of calcification is required. C-type lectin (calcium dependent lectin group) receptors are involved in immunity by contributing to the recognition of pathogens through the binding of glycoproteins [38], where binding specificity varies according to differences in glycosylation [11,39]. The up-regulation of C-type lectin receptor in *P. pelagicus*, observed pre- and post-molt, is consistent with providing anti-calcification properties, by way of glycoprotein affinity, to uncalcified cuticle. We propose that in addition to its role in immunity, the C-type lectin receptor recognises and attaches to endogenous glycoproteins in the cuticular matrix based on their glycosylation patterns, facilitating the inhibition of calcification.

**Figure 2:**
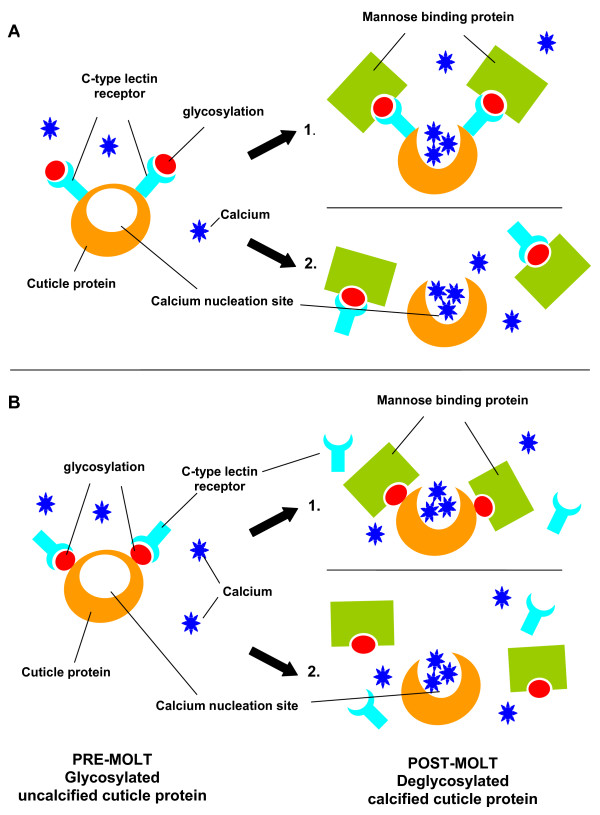
**Alternative conceptual models depicting the proposed involvement of C-type lectin receptors and mannose binding protein in the regulation of calcification**. A C-type lectin receptors bind to the cuticle protein thus facilitating glycosylation. Mannose binding protein binds to glycosylated regions thereby exposing the calcium nucleation site and enabling calcification, by either conformational change (A1) or deglycosylation (A2). B C-type lectin receptor binds to the sugar moieties of the glycosylated cuticle proteins, masking the calcification nucleation site. Competitive binding by the mannose binding protein to the glycosylation sites facilitates calcification exposing the calcium nucleation site either through conformational change to the cuticle protein (B1) or by deglycosylation (B2).

The expression profile of the lectin, mannose binding protein, on the other hand, was up-regulated by a factor of 8.8-fold only in the post-molt stage when compared to intermolt, and was not differentially expressed in any other molt stage (Figure 1). This up-regulation, specific only to the post-molt period, at the time in which calcification of the cuticle occurs, points to the possibility that the mannose-binding protein may directly facilitate calcification. If glycosylation of cuticular proteins inhibits calcification, and, as deglycosylation of cuticular proteins (via lectin affinity assays) has been observed post ecdysis to coincide with the calcification of the exoskeleton [16-18,20], the presence of a lectin such as mannose-binding protein during post-molt may facilitate calcification via deglycosylation of the cuticular proteins. Our hypothesis is that C-type lectin receptors either facilitate the glycosylation of cuticular proteins, or bind to the sugar moieties of the glycosylated cuticle proteins themselves, thus preventing calcium salts from entering the calcium nucleation site. Following ecdysis, the mannose-binding protein either binds to the mannose rich regions of the glycosylated cuticular proteins, or through competitive binding dislodges the C-type lectin receptor that is attached to the glycosylated cuticular proteins, thereby causing conformational changes to the glycoprotein, or facilitating deglycosylation, thus freeing the calcium nucleation sites and enabling the process of biomineralization to occur. A diagrammatic representation of this proposed model of calcification regulation is depicted in Figure 2. This hypothesis is consistent with *in-vitro *lectin binding assays that report the deglycosylation of cuticular proteins post ecdysis [16-18,20].

POs in insects and crustaceans participate in innate immunity, pigmentation, wound healing and repair of the damaged exoskeleton, as well as hardening (via sclerotization) of the exoskeleton after molting [9,23]. PO is activated by a serine protease activation cascade [23]. With relation to the immune system, this activation of zymogenic proPO into active PO is triggered by the presence of minute amounts of compounds of microbial origins such as β-glucans, lipopolysaccharides, and peptidoglycans [23]. These compounds are recognised by pattern recognition receptors. A pathway involved in innate immunity, the lectin pathway, uses a pattern recognition receptor, the mannose-binding lectin, which upon recognition of an infectious agent, triggers the activation of the lectin-complement pathway, through mannose-binding lectin associated serine proteases (members of the serine protease superfamily) [40,41]. Prominent ligands for mannose binding lectin are D-mannose and N-acetyl-D-glucosamine [40]. Components of this pathway have been isolated from ascidians, lampreys, frogs and mammals [40]. Lectins (carbohydrate-binding proteins), thus, play an important role in innate immunity by recognising the glyco-profiles of a wide range of pathogens and triggering PO activation [11,40]. Considering the involvement of POs in the sclerotization of the arthropod cuticle, the role of mannose binding protein in the activation of serine proteases, and its specific up-regulation exclusively during post-molt, we suspect that the glycosylation of cuticle proteins and their recognition by mannose-binding protein, may also participate in the PO activation pathway, leading to sclerotization of the cuticle in addition to its proposed role in calcification described above.

Trypsin and chymotrypsin also belong to the serine protease superfamily. Like most proteolytic enzymes, both are synthesized as inactive zymogen precursors (trypsinogen and chymotrypsinogen) to prevent untimely enzyme activity [42]. Trypsin and chymotrypsin are structurally very similar, although they recognise different substrates. Trypsin acts on lysine and arginine residues, while chymotrypsin acts on large hydrophobic residues such as tryptophan, tyrosine and phenylalanine [43]. Trypsin was shown to directly activate PO in the cuticle of the lobster *Panulirus argus *[44]. The authors also tested the intrinsic activity of lobster PO in the cuticle, throughout the molt cycle. The inactive forms of PO increased steadily from post-molt through to the early pre-molt stage, while the highest level of intrinsic activation of PO in the cuticle of lobsters was observed in late pre-molt [44]. Molt cycle-related differential expression of trypsin-like and chymotrypsin-like cDNAs, was also observed in our study. Trypsin-like transcripts were found to be 6.2-fold up-regulated in late pre-molt compared to ecdysis, and 8.2-fold up-regulated in intermolt compared to post-molt (Figure 1). Chymotrypsin-like transcripts however, were up-regulated (5-fold) only in intermolt when compared to post-molt. The lack of chymotrypsin-like up-regulation in late pre-molt indicates a functional difference in PO activation with respect to trypsin-like, indicating that different proPOs may be involved in the sclerotization process in the crustacean cuticle. The up-regulation of trypsin-like transcripts in pre-molt and intermolt mimics the expression pattern of a serine protease prophenoloxidase-activating factor (PPAF), discovered in the cuticle of *C. sapidus *[25]. High transcript levels of PPAF were observed in *C. sapidus *at the pre-molt stage, levels dropped at ecdysis and began to increase again 24 hours after ecdysis [25]. These data suggest that serine protease mediated PO activation is molt cycle-related, and is up-regulated in intermolt and prior to molting. The up-regulation of trypsin-like and chymotrypsin-like transcripts in the intermolt period compared to post-molt observed here, suggests that they may be required for cuticle sclerotization in the final stages of hardening, as proposed for PPAF in *C. sapidus*. The up-regulation of trypsin-like in the late pre-molt period compared to ecdysis, implies that the enzyme is either synthesised and remains in an inactive form before it is required for cuticle sclerotization post ecdysis, or that it may activate POs, before the old exoskeleton is shed, to promote melanization (pigment deposition) in the new cuticle.

Hemocyanin is phylogenetically related to POs, however unlike POs, hemolymph hemocyanin is not able to catalyse the conversion of monophenols to diphenols, but is only able to covert diphenols to o-quinones [22]. Eight distinct transcripts of hemocyanin displayed molt cycle-related differential expression in this study, five of these correspond to subunits 1–3 and 5, previously isolated from *C. magister*. Hemocyanin has been located in the cuticle of crustaceans; it was detected in the cuticle of the prawn *Penaeus japonicus *during the intermolt and post-molt stages, however not in the pre-molt stage [28]. In our study hemocyanin transcripts were up-regulated in the late pre-molt stage (combined average of 6.8-fold) when compared to ecdysis, and in the intermolt period by 8.2-fold when compared to post-molt (Figure 1). Given that there is a temporal delay between the synthesis of hemocyanin in the hepatopancreas and its subsequent deposition in the cuticle, the up-regulation of hemocyanin transcripts in the late pre-molt period is likely to precede its incorporation in the cuticle during post-molt. Similarly, the length of the intermolt period, and the continued synthesis and/or repair of the exoskeleton into the intermolt stage, supports the premise that up-regulation of hemocyanin transcripts in intermolt occurs prior to the incorporation of hemocyanin into the intermolt cuticle. The enzymatic activity of cuticular hemocyanin has been demonstrated to be higher than that of hemocyanin from the hemolymph, and capable of catalysing both consecutive PO reactions: the o-hydroxylation of tyrosine and the oxidation of dihydroxyphenylalanine (DOPA) to dopaquinone [28]. The molt cycle-related transcript expression profile established in this study, together with the protein localisation and enzyme activity of hemocyanin [28], are indicative of the importance of hemocyanin mediated PO activity in the exoskeletal sclerotization process.

Proteins involved with the crustacean coagulation cascade, have been linked to PO activation [32]. An experiment describing the functional activation (without proteolytic cleavage) of PO activity in hemocyanin by a clotting enzyme in the horseshoe crab [32], suggests that the binding of the clotting protein may lead to a conformational change in hemocyanin allowing large phenolic compounds to enter the active site, thus promoting PO activity. Treatment of hemocyanin with SDS also enables non-enzymatic PO activation via conformational change [45]. In our study we observed that transcripts of a clotting protein precursor-like gene were up-regulated by 5.6-fold in the intermolt stage when compared to post-molt, and by 7.6-fold in late pre-molt compared to ecdysis (Figure 1). These data show that clotting protein precursor-like is expressed in the intermolt and pre-molt period, and is analogous to the expression profile of hemocyanin described above. The concomitant up-regulation of hemocyanin and clotting protein precursor-like gene during the molt cycle of *P. pelagicus*, together with their associated PO activity, suggests that these compounds play a role in the sclerotization and/or melanization of the crustacean exoskeleton.

The putative antimicrobial peptide carcinin-like was also up-regulated in the intermolt stage (3.4-fold) when compared to post-molt, and by 8.8-fold in late pre-molt compared to ecdysis (Figure 1). Antimicrobial peptides are also able to induce the intrinsic PO activity of hemocyanin in horseshoe crabs [31]. Furthermore, antimicrobial peptides have been isolated from the cuticle of the horseshoe crab [46], suggesting that they may facilitate the sclerotization of the cuticle, in addition to acting as antimicrobial substances. In fact other potential PO activator transcripts that have also been associated with immunity such as trypsin-like and clotting protein precursor-like as discussed above, also follow this expression profile.

## Conclusion

Biomineralization is, at least partly, controlled by the organic matrix itself, in which the three dimensional arrangement and overall composition of the entire matrix determines the function at any given time [13]. Organic matrices are thought to regulate biomineralization in a variety of ways: by initiation of crystal growth, determination of crystal polymorph, control of crystal shape and termination of crystal growth [47]. Glycosylation of cuticular proteins has been found to affect their calcification at critical periods during the molt cycle [16,21]. We have demonstrated that the C-type lectin receptor and mannose binding protein display molt cycle-related differential expression profiles, and propose possible regulatory functions for these proteins in the calcification of crustacean cuticle. Additionally the up-regulation of PO inducers such as trypsin-like, clotting protein precursor-like and antimicrobial proteins, in conjunction with hemocyanin, suggests that multiple PO activation pathways may modulate the sclerotization cascade in the exoskeleton of *P. pelagicus*. The expression of these activators in the intermolt and pre-molt stages, before ecdysis occurs, suggests that they are synthesised prior to their utilization, and remain in an inactive form until they become "triggered", perhaps via a change in glycosylation, to activate the PO cascade. In addition to their proposed role in the calcification of cuticular proteins, C-type lectin receptor and mannose-binding protein may also regulate the activation of the PO pathway by virtue of their affinity for glycoproteins.

## Methods

### Animal selection

*P. pelagicus *crabs were supplied by staff at the Department of Primary Industries & Fisheries (DPI&F) Bribie Island Aquaculture Research Centre (BIARC). The crabs were individually housed in a flowthrough system at an ambient water temperature of 24°C, and fed a commercial diet (Ebistar, Higashimaru, Japan) twice daily. Two size groups of crabs were used, small crabs of an average carapace width of 4 cm, and larger crabs of an average carapace width of 11 cm. All crabs were molt staged by examination of pleopod paddles for epidermal retraction and grouped into the following molt stages; molt (shedding of the exoskeleton), post-molt (pliable exoskeleton), intermolt (hard exoskeleton with no evidence of epidermal retraction) early and late stage pre-molt (based on the extent of epidermal retraction) [48].

### cDNA library construction

Two cDNA libraries were constructed using various source tissues, selected in order to provide a diverse collection of transcripts, and representing a broad range of tissue functions and physiological states in all molt stages. One of the cDNA libraries was synthesised from whole animals in order to obtain transcripts from each tissue type. For this library, six small crabs, from each of the following five molt stages; molt, post-molt, intermolt, early and late pre-molt stages, were selected, snap frozen and ground under liquid nitrogen. The other cDNA library, was derived from organs previously identified as being important to the molt cycle of crustaceans and served to enrich the array with sequences particularly relevant to crustacean molting. The tissues represented in the *P. pelagicus *organ library were brain, eyestalk, mandibular organ (MO) and Y-organ. These tissues were obtained from six anaesthetised large *P. pelagicus *crabs from each of molt, post-molt, intermolt, and early and late pre-molt stages, and stored in RNA later (Ambion, Austin, USA).

Total RNA was purified from each tissue sample using TRIZOL reagent as recommended by the manufacturer (Invitrogen Life Technologies, Carlsbad, CA, USA). Concentration and purity of the RNA were determined using a spectrophotometer (GeneQuant Pro, GE Healthcare UK Ltd., Buckinghamshire, England) with 260 and 280 nm readings. RNA quality was assessed for all samples by visualisation on a denaturing formaldehyde RNA gel (protocol recommended by Qiagen, Valencia, CA, USA) and ethidium bromide staining. Each cDNA library was constructed by pooling equal amounts of total RNA from all molt cycle stages.

A commercial cDNA library synthesis system (SMART cDNA library construction kit, Clonetech, Mountain View, CA, USA) was used for the construction of each library according to the manufacturer's instructions. Only the final cloning step was modified so that instead of using the λ TriplEx2 vector supplied with the kit, the size fractionated cDNA was ligated into pGEM-T Easy (Promega, Madison, WI, USA) as per manufacturer's instructions, and transformed into XL10 Gold ultracompetent cells (Stratagene, La Jolla, CA, USA) according to the manufacturer's protocol. 80 clones, randomly selected from each library, were then sequenced and analysed using BLAST  to determine gene redundancy. The primer used for sequencing was the 5'SMARTlibPCR primer (5'-AAGCAGTGGTATCAACGCAGAGT-3') a modification of the SMART IV oligonucleotide supplied with the SMART cDNA library construction kit (Clonetech).

### Screening for redundant clones

Upon examination of 160 clones, from the cDNA libraries of both whole crab and crab organ, redundancies for 16S ribosomal RNA (rRNA) genes were found to be as high as 30%. To remove 16S rRNA carrying plasmids, all of the clones were first screened for the 16S rRNA sequence, using a colony hybridisation method [49]. Briefly three probes, (500 bp, 344 bp and 300 bp in length) were designed from separate regions of the 16S rRNA sequence. These probes were PCR amplified and labelled with ^32^P, then hybridised to clones that had been fixed to nitrocellulose filters. Following an overnight incubation at 55°C in hybridisation buffer (6× SSC and 1% SDS), the filters were washed twice at 55°C in a solution of 6× SSC and 0.2% SDS for 30 min, sealed within plastic and exposed onto autoradiography films (GE Healthcare UK Ltd.) at -70°C using intensifying screens. The films were then developed according to supplier's instructions.

### Construction of custom *P. pelagicus *cDNA microarrays

5000 unsequenced clones, that had been pre-screened for 16S ribosomal RNA, were randomly selected for spotting onto the microarray slides. 2400 were selected from the whole crab library and 2600 from the crab organ library. These were grown overnight in LB containing 50 μg/ml ampicillin. The clones were sent to the AgGenomics (Bundoora, Vic, Australia) microarray printing facility. The clones were PCR amplified using kit-supplied primers (Clontech) and contact-spotted (in duplicate) using pins, onto amino silane coated glass slides, in a 50% DMSO buffer. Known crab genes, which were identified at the initial sequencing stage, such as actin [GenBank:EF110528], cryptocyanin [GenBank:EF102021], hemocyanin [GenBank:EF110534], metallothionein [GenBank:EF110529], opsin [GenBank:EF110527] and ubiquitin [GenBank:EF110526] were spotted onto the arrays for use as controls. Genes specifically associated with the molting process such as molt-inhibiting hormone (MIH) [GenBank:EF110524], crustacean hyperglycaemic hormone (CHH) [GenBank:EF110525] and farnesoic acid methyl transferase (FaMeT) long isoform [GenBank:DQ085282] [50], were isolated separately from *P. pelagicus *through the design of gene specific primers and spotted on to the arrays. In addition universal reference RNA standard controls (Lucidea, GE Healthcare UK Ltd.) were also spotted onto each array, as were negative control spots of 50% DMSO (without cDNA). The cDNA was bound to the slide surface by baking and UV crosslinking.

### Experimental Design

In order to identify differential gene expression across molt stages, two consecutive molt stages were compared on each array in a dual colour (Cy3 and Cy5) experiment. RNA samples were pooled across subjects in order to reduce the effect of biological variation. A formula, which dictates the total number of subjects and arrays required for the pooled experiment to obtain gene expression estimates and confidence intervals comparable to those obtained from a non-pooled experiment [51], gave 90% confidence if nine subjects were pooled across a total of three arrays. To this effect, equal amounts of total RNA from three crabs in one molt stage, were pooled, and compared against equal amounts of total RNA pooled from three crabs in another molt stage, on one array. This was repeated three times in total, the different molt stages were labelled with Cy3 or Cy5 respectively. Consecutive molt stages were compared in the following format; post-molt (Cy3) with intermolt (Cy5), intermolt (Cy3) with early pre-molt (Cy5), early pre-molt (Cy3) with late pre-molt (Cy5), late pre-molt (Cy3) with ecdysis (Cy5), and ecdysis (Cy3) with post-molt (Cy5).

Technical variation, that is array-to-array variability, in these microarray experiments, was addressed through spot duplication. Two identical grids consisting of each amplified cDNA and including the controls described above were printed onto the left and right sides of each horizontally orientated array, thus affording spatial separation between duplicate spots, to allow for the normalisation of potential hybridisation anomalies.

### Microarray Hybridisations

Nine small crabs (six of these were also used in the above described whole crab cDNA library construction) were snap frozen, ground under liquid nitrogen and RNA was isolated using TRIZOL reagent as recommended by the manufacturer (Invitrogen Life Technologies). The RNA was DNase treated using RQ1 RNase free DNase (Promega) as per manufacturer's instructions and purified using RNeasy Mini Kit (Qiagen) as recommended by the manufacturer. RNA quality was assessed by visualisation on a denaturing formaldehyde RNA gel (protocol recommended by Qiagen) using ethidium bromide staining. Concentration and purity of the RNA were determined by measuring the absorbance at 260 nm and 280 nm using a spectrophotometer (GeneQuant Pro). One microgram of Lucidea universal RNA control (GE Healthcare) was added to 10 μg of pooled total RNA for each molt stage sample, the RNA was converted to cDNA then labelled and hybridised to the array using the 3DNA Array 900 MPX expression array detection kit (Genisphere Inc., Hatfield, PA, USA) according to the manufacturer's protocol. Briefly, RNA was reverse transcribed using a random primer combined with an oligo dT primer. The RNA was then degraded and the cDNA tailed with dTTP followed by ligation to a dendrimer-specific capture oligo (specific for either Cy3 or Cy5). Microarray slides were denatured prior to use by immersion in 95°C MilliQ water for 5 minutes, the slides were then transferred to 95% ethanol at room temperature for 2 minutes. Slides were spun dry to reduce streaking at 800 RPM for 2 minutes. The Cy3 and Cy5 "tagged" cDNAs were combined and then hybridised to the array by overnight incubation in a humidity chamber at 65°C using the kit supplied non-formamide SDS-based buffer and a poly T based blocker, as per manufacturer's specifications. The "tagged" cDNA was washed with a series of three SSC-based buffers, the first wash occurred at 65°C for 15 min, the other wash steps were carried out at room temperature for 10 min each. The slides were spun dry at 800 RPM for 2 minutes. Fluorescent 3DNA capture reagent (which carries a sequence complementary to the Cy3 and Cy5 tag) was then hybridised to the array using the SDS-based buffer with added Anti-Fade reagent (inhibits photobleaching of Cy 5) at 65°C for four hours. The fluorescent reagent was then washed as described above for the cDNA hybridisation.

### Data analysis

Microarray slides were scanned using a white-light ArrayWorx Biochip Reader (Applied Precision, LLC, Issaquah, Washington, USA). ImaGene (BioDiscovery Inc., El Segundo, CA, USA) was then used to process images and create spot intensity reports, while CloneTracker (Biodiscovery Inc.) was used to generate gene ID mapping files and assign gene identification. Final intensity reports were retrieved as raw spot intensities in tab-delimited files. The data set is deposited in the Gene Expression Omnibus (GEO) database (accession no. GSE6997) at the following site: .

Microarray data analysis was performed by Emphron Informatics (Chapel Hill, Qld, Australia). Briefly, data was normalised using the robust scatter plot smoother LOESS (also known as "LOWESS" for locally-weighted regression and smoothing scatter plots) [52]. For each chip, normalisation was applied to the left and right sides separately (spatial positioning of clones spotted in duplicate was in the format of two grids located on the left and right side of each array when orientated horizontally). Individual microarray quality was assessed using M vs A scatter plots. M-A plots were constructed for each slide, where the log-intensity ratio M = log(Cy3/Cy5) = [logCy3 - logCy5] were plotted against the mean log-intensity A = [(logCy3 + logCy5)/2] as described by [53]. Potential dye intensity biases in the microarray data sets were assessed by examining the back-to-back histograms of Cy3 and Cy5 expression. Since each feature is spotted onto an array in duplicate, and three biological replicates are performed per molt stage comparison, a standard error, a t-statistic, and t-distribution (P value) can be calculated for each feature represented on the array. Standard errors were based on the mean of technical replicates for a given slide. For each of the features on the slide, a P value for differential expression was calculated using the empirical Bayes procedure [54]. These P values were then adjusted using the False Discovery Rate (FDR) procedure [55]. This conservative procedure provides control of the Family-Wise Error Rate (FWER), which is the probability of at least one false positive. The advantage of controlling the FWER is that any features identified as differentially expressed are highly likely to be so, however, the disadvantage is that it is easier to omit features that are differentially expressed. Differential gene expression was only considered significant if the (FDR) adjusted P value was less than 0.05. These genes are listed in the results section where M is the log_2 _fold change in expression and t is the t-statistic. Positive values of M and t indicate up-regulation in the Cy3 sample whereas negative values of M and t indicate down-regulation in the Cy3 sample. Genes were considered up- or down-regulated when the logarithm of the gene expression ratio (M) was more than 1 or less than -1, that is there was a 2-fold (or greater) difference in expression levels.

### Sequence and phylogenetic analysis

Following hybridisation experiments, clones which displayed differential expression (P ≤ 0.05) patterns across molt stages were sequenced. Overlapping sequences (contigs), that likely represent the same cDNA, and clones without sequence identity to other cDNAs (singlets) were identified by comparing all sequences against one another in sequencher (Gene Codes Corporation, Ann Arbor, MI, USA). The genes were annotated with the name of the highest basic local alignment search tool (BLAST) [56] score from an analysis of GenBank entries by the BLASTx and BLASTn procedures. Protein domains were identified from the Pfam database [57], and InterProScan .

## Authors' contributions

AVK contributed to the conception and design of the project, analysis and interpretation of the data. AVK also carried out the molecular studies and drafted the manuscript. AE contributed to the conception and design of the project, analysis and interpretation of the data and drafted the manuscript.
